# Long-term trajectories of peak expiratory flow rate in older men and women show linear decline mainly determined by baseline levels

**DOI:** 10.1007/s40520-024-02735-5

**Published:** 2024-04-16

**Authors:** Natasja M. van Schoor, Renate T. de Jongh, Paul Lips, Dorly J. H. Deeg, Almar A. L. Kok

**Affiliations:** 1 Epidemiology and Data Science, Amsterdam UMC Location Vrije Universiteit Amsterdam, De Boelelaan 1117, Amsterdam, The Netherlands; 2 Aging & Later Life, Amsterdam Public Health Research Institute, De Boelelaan 1117, Amsterdam, The Netherlands; 3 Department of Endocrinology and Metabolism, Amsterdam UMC Location Vrije Universiteit Amsterdam, De Boelelaan 1117, Amsterdam, The Netherlands

**Keywords:** Peak expiratory flow rate, Trajectories, Ageing, Community-dwelling

## Abstract

**Background:**

Peak expiratory flow rate (PEFR) predicts mortality and other negative health outcomes. However, little evidence exists on how PEFR changes with ageing and how trajectories of change differ among older people.

**Aims:**

To identify trajectories of PEFR in older men and women, and to study characteristics associated with these trajectories.

**Methods:**

Data from the Longitudinal Aging Study Amsterdam were used, an ongoing cohort study in a representative sample of Dutch older men and women. PEFR was assessed using the Mini-Wright peak flow meter across a 13-year follow-up in 991 men and 1107 women. Trajectories were analyzed using Latent Class Growth Analysis.

**Results:**

Mean age was 72.5 (SD 8.4) in men and 72.4 (SD 8.4) in women. In men, three declining trajectories were identified, i.e. high, intermediate and low, with prevalences of 30%, 46% and 24%, respectively. In women, two declining trajectories were identified, i.e. high and low, with prevalences of 62 and 38%. All trajectories showed linear decline and differed mostly with regard to their intercept. Significant differences between trajectories with regard to baseline demographic, health and lifestyle characteristics were observed, e.g., men and women in the low PEFR trajectory were older, had more chronic diseases, and were more often smoker.

**Discussion and conclusions:**

Trajectories in both men and women differ mainly in baseline level of PEFR and not in rate of decline over time. Therefore, one PEFR measurement might be sufficient to give an indication of the trajectory that an older adult is likely to follow.

## Introduction

Several studies have shown that lower lung function is an important predictor of all-cause and disease-specific mortality [1–4]. Lung function declines during ageing, and the prevalence of lung conditions, such as chronic obstructive pulmonary disease and lung infections, sharply increases with age [5].

The decline in lung function during ageing was confirmed in a recent systematic review [6]. Most studies in this review used Forced expiratory volume in 1 s (FEV_1_) and Forced Vital Capacity (FVC) to assess lung function as these are the gold standard in the clinic. In addition, two studies assessed lung function using peak expiratory flow rate (PEFR) [7, 8]. PEFR is defined as a person’s maximum speed of expiration and shows a strong correlation with FEV_1_ (r = 0.83) [9]. It primarily reflects large airway flow and depends on the amount of airway obstruction, the voluntary effort and muscular strength of the patient. Advantages of PEFR are that it is an inexpensive method, easy to measure, and less of a burden to the patient. Therefore, it might be an attractive indicator for health status when spirometry (FEV_1_, FVC) is not available or feasible [2].

In 2015, the World Health organization proposed to monitor the intrinsic capacity of older adults with the aim to maintain and enhance healthy ageing [10]. Respiratory function was suggested to be a feasible marker of one of the domains of intrinsic capacity, i.e. the vitality domain [11, 12]. As this proposal is intended for worldwide implementation, PEFR might be a suitable marker for assessing the respiratory function of older adults. However, before developing a monitoring strategy, it is of importance to study the rate of change in PEFR during normal ageing. In addition, it is important to study whether subpopulations with different rates of change during ageing can be identified. Differences in rates of change, also termed trajectories, can be examined using data-driven methods such as mixture modelling. These trajectories may be characterized by different intercepts, slopes and growth parameters (e.g. linear or curvilinear). For example, one subpopulation may start with a higher lung function (i.e. a difference in intercept) and/or show a stronger decline (i.e. a difference in slope) than another subpopulation. Subpopulations with stronger decline may be characterized using demographic variables (e.g. lower socio-economic status), health variables (e.g. having more chronic diseases) and lifestyle variables (e.g. smoking). The aim of the current study is to identify subpopulations with distinct trajectories of PEFR in men and women aged 61 years and over. As a second aim, we examine how these subpopulations differ on demographic, health and lifestyle characteristics.

## Methods

### Design and study sample

Data from the Longitudinal Aging Study Amsterdam (LASA) were used. LASA is an ongoing multidisciplinary cohort study on predictors and consequences of changes in physical, cognitive, emotional and social functioning in older persons. A random sample of men and women aged 55 years and over, stratified by age, sex, urbanization grade and expected 5-year mortality rate was drawn from the population registers of eleven municipalities, in three regions of the Netherlands. In total, 3107 persons were enrolled in the baseline examination in 1992/93 [13, 14]. Measurement cycles were repeated every 3–4 years, and included a general interview and a medical interview. PEFR was assessed in the medical interview. For the current study, 1995/96 was taken as the baseline as some of the characteristics potentially associated with PEFR trajectories were only assessed in 1995/96. Follow-up PEFR measurements were performed in 1998/9, 2001/2, 2005/6 and 2008/9. For the current study, persons with at least one PEFR measurement were selected (n = 991 men; n = 1107 women, age range 61–100 years). The Institutional Review Board of the VU University Medical Center approved the study, and all persons gave informed consent.

### Peak expiratory flow rate (PEFR)

PEFR is defined as a person’s maximum speed of expiration. It primarily reflects large airway flow. PEFR was assessed using the Mini-Wright peak flow meter. For the measurements, the subjects were asked to take a maximum inspiration and to breathe out with maximum effort into the peak flow meter. The highest score of three measurements in millimeters (ml) was used [15].

### Age and sex

Data on age and sex were derived from population registries. All analyses were stratified on sex because of the differences in lung function between men and women [16].

### Characteristics

Potential characteristics of participants with different PEFR trajectories were selected largely based on the lung function literature [6, 16]: educational level, weight, height, number of chronic diseases, chronic obstructive pulmonary disease (COPD), corticosteroid use, smoking, physical activity, and C-reactive protein (CRP). We also studied serum 25-hydroxyvitamin D [25(OH)D] [17], physical performance [3], and grip strength [3] as these were related to PEFR in earlier studies from our group. In addition, having a partner, perceived self-efficacy, the presence of pet(s) in the household and the presence of dog(s) in the household were examined. Persons with a partner may be more healthy than persons without a partner [18]. Perceived self-efficacy may influence PEFR outcomes, as this may reflect the effort that one expends to perform the test. The hairs of pets, i.e. all pets including dogs, might have a negative effect on PEFR, while walking one’s dog might have a positive effect on lung function.

Educational level was self-reported as the highest education level completed. This was converted into elementary school or less, secondary school, or higher education. Body weight was measured without clothes and shoes using a calibrated bathroom balance scale. Body height was measured using a stadiometer. Number of chronic diseases was assessed by self-report of seven major diseases, i.e. COPD, cardiac disease, peripheral arterial disease, diabetes mellitus, stroke, cancer, and joint disorders (osteoarthritis/rheumatoid arthritis). Ever use of corticosteroids for longer than 3 months, i.e. prednisone, cortisone, decadron, was assessed by self-report. Smoking (never /stopped smoking > 15 years ago, former /stopped smoking <  = 15 year ago, current) was assessed by self-report. Physical Activity was assessed in minutes per day by the LASA Physical Activity Questionnaire, a validated questionnaire covering walking outside, bicycling, gardening, light household activities, heavy household activities, and a maximum of two sport activities during the previous two weeks [19]. Serum 25-OHD was determined using a competitive protein binding assay (Nichols Diagnostics, San Juan Capistrano, CA, USA). The interassay coefficient of variation was 10%. CRP was determined using a sandwich type ELISA in which polyclonal rabbit anti-CRP antibodies were used as catching antibodies and a biotinylated mAb against CRP (CLB anti-CRP-2) as the detecting antibody. The interassay coefficient of variation was less than 4.2%. Physical performance was assessed by three tests: time needed to walk three meters along a line, turn 180 degrees and walk back (walking test); time needed to stand up from and sit down on a chair five times with arms folded across the chest (chair stands test); and the ability to perform the tandem stand (one foot placed behind the other on a straight line) for at least ten seconds (tandem stand) (adapted from [20]). For the walking test and chair stands test, scores one to four were given according to the quartile of the distribution of time needed with score one representing the worst quartile, i.e. the longest time, and score four the best quartile, i.e. the shortest time. Score zero was given to those respondents who could not complete the test. The tandem stand was categorized as: unable (score 0), able to hold position for 3–9 s (score 2), and able to hold position for at least 10 s (score 4). Total physical performance sum score ranges from zero to 12 with 12 indicating good physical performance. Handgrip strength was assessed using a strain-gauged dynamometer (Takei TKK 5001, Takei Scientific Instruments Co. Ltd, Tokyo, Japan). Participants were asked to perform two maximum force trials with each hand, while standing with their arm along the body. To calculate the total score, the maximum values of the right and the left hand were summed, and divided by two [21]. If only one hand could be used, the maximum value of that hand was taken. Perceived self-efficacy was assessed using the 12-item version of the General Self-Efficacy Scale [22]. Total score ranges from 12 to 60 with higher scores indicating higher perceived self-efficacy. Pet(s) in the household (yes/no) and dog(s) in the household (yes/no) were assessed by self-report.

### Statistical analyses

Baseline characteristics of men and women were presented using descriptive statistics. Trajectories of PEFR were analyzed for men and women separately using Latent Class Growth Analysis (LCGA) in Mplus 8.7 (Muthén & Muthén, 2021). LCGA is a longitudinal technique and an extension of conventional growth modelling. Conventional growth modelling assumes that one (average) trajectory adequately describes the developmental pattern of the entire sample. In LCGA individuals do not need to come from one single underlying population, but can come from multiple, unobserved (or latent) subpopulations, which each have their own growth parameters (intercept, slope) and means. Using LCGA, for each individual a growth curve is estimated taking into account the individual PEFR measurements, which all have the same weight. The intercept and slope are used to assign individuals to different subpopulations. LCGA identifies the number, size, and (latent) growth parameters of these underlying subpopulations, also called classes.

Age was used as the time metric for the trajectories of PEFR. For this, the data that was originally structured according to measurement wave was restructured to represent observed PEFR values at 3-year age intervals, covering intervals ranging from 61 to 64 years to 97–100 years. Missing data was handled using Full Information Maximum Likelihood estimation, under the assumption of Missing At Random. To determine the optimal number of latent classes (subpopulations), we considered multiple criteria. First, the Bayesian Information Criterion (BIC). The BIC is commonly used with LCGA, considering both the likelihood of the model and the number of parameters in the model; a lower BIC value indicates a better model fit. A decrease of at least 10 points usually denotes a statistically significant improvement. Second, we considered the Bootstrapped Likelihood Ratio Test (BLRT; a p-value < 0.05 is interpreted as better fit of the model with *k* classes compared to the model with *k-1* classes). Third, we considered the entropy, which is a measure for classification accuracy; entropy values closer to 1 indicate higher accuracy. Fourth, in choosing the optimal number of classes we accepted only latent classes including at least 5% of the sample, and took into account clinical interpretability.

In a sensitivity analysis, we also included body height in the model to model the trajectories and to examine whether this affected the number of classes and model fit, as a higher body height is correlated with a higher PEFR [23].

Finally, we estimated the associations between characteristics and latent class membership by assigning individuals to the class with their highest probability, and exporting this to SPSS 28.0.1.1 (IBM SPSS Statistics, 2022). Descriptive statistics were used to compare the characteristics between classes. Differences were tested using independent T-test and One-Way ANOVA for continuous characteristics and Chi-square test for categorical characteristics. Next, the association between the characteristics and classes were adjusted for baseline age and education using multinomial regression in men and logistic regression in women. Age is a well-known risk factor for decline in lung function, and educational level was used as a proxy for low socio-economic status. Earlier research showed that older adults with disadvantaged early-life socioeconomic circumstances showed lower levels of PEFR compared with those with advantaged early-life socioeconomic circumstances [24].

## Results

### Baseline characteristics

In Table 1, baseline characteristics of men (n = 991) and women (n = 1107) are presented. The mean baseline age was 72.5 years in men and 72.4 years in women. In men, 15.4% reported to have COPD versus 11.0% in women. Furthermore, 25.7% of men reported to be a current smoker versus 13.2% of women. The baseline PEFR was 423.2 ml (SD: 134.2) ml in men and 325.6 ml (SD: 96.7) in women. In total, 5413 PEFR observations were included in the analyses. The median number of waves was 3 per person.

**Table 1:** Baseline characteristics of the study sample

Baseline variables	Men (n = 991)^a^	Women (n = 1107)^a^
Age (years)	72.5 ± 8.4	72.4 ± 8.4
Partner (% yes)	80.8	48.4
Educational level (%)		
Lower	28.2	50.5
Medium	53.9	41.2
Higher	17.9	8.2
Height (cm)	173.0 ± 6.8	160.1 ± 6.4
Weight (kg)	78.0 ± 11.9	71.0 ± 13.0
Chronic obstructive pulmonary disease (% yes)	15.4	11.0
Number of chronic diseases (%)		
0	34.1	28.5
1	34.4	38.7
2 or more	31.6	32.7
Medication use (%)		
Ever used corticosteroids > 3 months (%)	3.9	5.1
Smoking (%)		
Never or > 15 years ago	52.0	78.4
Former	22.3	8.4
Current	25.7	13.2
Physical activity (min/day)	107.1 [58.6–165.7]	171.4 [113.6–238.9]
Serum 25(OH)D (nmol/L)	52.8 ± 18.8	45.7 ± 17.9
CRP (µg/ml)	3.3 [1.6–6.7]	3.1 [1.4–6.5]
Physical performance (range 0–12)	8.3 ± 3.0	7.2 ± 3.4
Grip strength (kgf)	35.1 ± 8.4	20.7 ± 5.0
Perceived self-efficacy (range 12–60)	42.7 ± 5.2	41.2 ± 5.3
Pet(s) in the household (% yes) ^b^	29.8	24.5
Dog(s) in the household (% yes) ^c^	38.6	24.5
PEFR at baseline (ml)	423.2 ± 134.2	325.6 ± 96.7

*BMI* body mass index, *serum 25(OH)D* serum 25-hydroxyvitamin D, *CRP* C-reactive protein, *PEFR* peak expiratory flow rate

^a^Presented are the mean ± standard deviation, median [interquartile range] or valid percentage

^b^Pet(s) in the household including dogs

^c^Question only asked in persons having a pet

### Trajectories

**Fig. 1:**
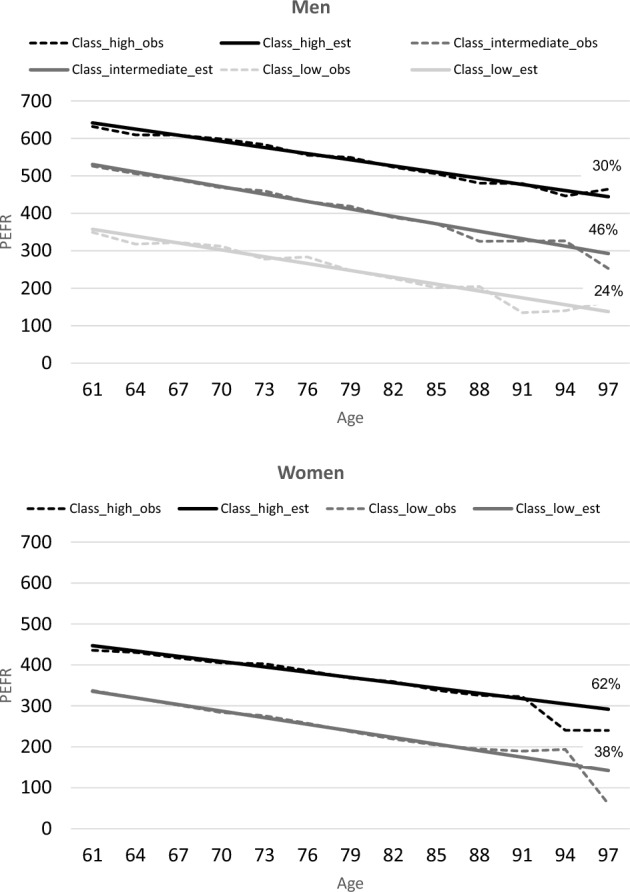
Trajectories of PEFR in men and women. *PEFR* Peak expiratory flow rate, *obs* observed values, *est* estimated values. Three classes were identified in men (high, intermediate, low) and two classes were identified in women (high, low). The prevalence of each class was presented as a percentage

In Table 2, the LCGA model fitting process in men and women are presented. Both in men and women, the BIC and entropy decreased with an increasing number of classes, while the BLRT p-value was no longer statistically significant at 4 classes in men and at 3 classes in women. In men, the entropy dropped from 0.75 at 3 classes to 0.73 at 4 classes; in women, the entropy dropped from 0.74 at 2 classes to 0.72 at 3 classes. For both men and women, the quadratic and cubic model did not lead to a significant decrease in BIC value. Based on these results, we decided to accept the linear 3-class solution in men (presented in bold in Table 2), which we will refer to as the high, intermediate and low PEFR trajectory, with prevalence rates 30%, 46% and 24%, respectively. In women, we accepted the linear 2-class solution (presented in bold in Table 2), which we will refer to as the high and low PEFR trajectory, with prevalence rates 62% and 38%, respectively. In Fig. 1, the trajectories in men and women are presented. PEFR decreased significantly with age in both men (slope (s) = −5.5/yr, p < 0.001 for high; s = −6.6/yr, p < 0.001 for intermediate; s = −6.1/yr, p < 0.001 for low) and women (s = −4.3/yr, p < 0001 for high; s = −5.4/yr, p < 0.001 for low). However, as can be observed in Fig. 1, trajectories in both men and women differed mainly in intercept (intercept (i) = 641.4, p < 0.001 for high; i = 530.5, p < 0.001 for intermediate; i = 357.5, p < 0.001 for low in men; i = 447.0, p < 0.001 for high; i = 335.5, p < 0.001 for low in women) and not much in the strength of decline over time.

**Table 2:** LCGA model fitting process in men (n = 991) and women (n = 1107)

	Men				Women			
Classes	SSA-BIC	Entropy	BLRT p-value	% in classes	SSA-BIC	Entropy	BLRT p-value	% in classes
	Linear model				Linear model			
**2**	29382	0.76	< 0.001	39**-**61	**33386**	**0.74**	** < 0.001**	**62-38**
**3**	**28942**	**0.75**	** < 0.001**	**30-46-24**	33162	0.72	0.2400	47**-**12-40
**4**	28822	0.73	0.1728	13**-**23-21**-**44	33010	0.72	0.0004	49**-**31-11**-**9
**5**	28736	0.71	0.0392	33**-**19-10**-**10-28	32973	0.67	0.2813	25**-**8-23**-**39-6
	Quadratic model				Quadratic model			
**2**	29386	0.76	< 0.001	39**-**61	33382	0.74	< 0.001	38**-**62
**3**	28937	0.75	< 0.001	24**-**30-46	33153	0.70	0.2986	43**-**16-41
**4**	28822	0.73	0.3616	14**-**20-23**-**43	33004	0.71	0.0014	9**-**12-49**-**31
**5**	28731	0.72	0.0495	34**-**28-19**-**9-11	32969	0.67	0.3549	22**-**7-26**-**6-39
	Cubic model				Cubic model			
**2**	29390	0.76	< 0.001	61**-**39	33389	0.74	< 0.001	62**-**38
**3**	28945	0.75	< 0.001	30**-**24-46	33159	0.72	0.1157	40**-**14-46
**4**	28834	0.73	0.4746	14**-**23-20**-**44	33012	0.71	0.0056	12**-**30-9**-**48
**5**	28745	0.72	0.0058	10**-**10-28**-**18-33	32982	0.67	0.3219	6**-**8-39**-**25-22

Accepted class solutions are depicted in bold

*SSA-BIC* Sample Size Adjusted Bayesian Information Criterion, *BLRT* Bootstrapped Likelihood Ratio Test

### Sensitivity analyses

Taking body height into account in the models led to similar results. In men, the linear 3-class solution showed the best fit with prevalence rates of 30%, 45% and 25%. In women, the linear 3-class solution had a significantly lower BIC, but the BLRT p-value was not significantly better (data not shown). We chose to accept the more simple solution, which was the linear 2-class solution with prevalence rates of 61% and 39%. Both in men and women, one of the trajectories of the 4-class solution had a prevalence rate < 5% (data not shown). The plots depicting the 3-class solution in men and the 2-class solution in women were highly similar to the main analyses (see supplementary Fig. 2 in the Appendix). The percentage agreement between the classes modelled without and with height was 96% in men and 97% in women. Therefore, further analyses on the characteristics were based on the classes found in the main analyses.

### Characteristics

Large, significant differences in baseline characteristics between trajectories were found in the univariable analyses for all characteristics, except for ever use of corticosteroids, and having a pet/dog in the household in men, and for weight and having a pet/dog in the household in women (see Appendix Tables 5 and 6). In Table 3, differences in baseline characteristics between trajectories in men are presented after adjustment for age and educational level. Men having a partner, having a higher physical performance or higher perceived self-efficacy had a lower odds for being in the intermediate as compared with the high PEFR trajectory. They also had a lower odds for being in the low as compared with the high PEFR trajectory. In addition, men having a higher weight had a lower odds for being in the low as compared with the high PEFR trajectory.

**Table 3:** Characteristics of intermediate and low PEFR trajectories in men: multinomial logistic regression adjusted for age and educational level

Characteristics	OR (95% BI) on intermediate versus high PEFR trajectory	OR (95% BI) on low versus high PEFR trajectory
Age (years)^a^	1.02 (1.00–1.04)*	1.04 (1.02–1.06)***
Partner (yes)	0.49 (0.30–0.78)**	0.33 (0.20–0.55)***
Educational level^b^		
Lower	2.28 (1.45–3.60)***	3.64 (2.09–6.35)***
Medium	1.31 (0.90–1.92)	1.81 (1.10–2.96)*
Higher	Reference group	Reference group
Height (cm)		
Q1	2.61 (1.48–4.60)***	5.29 (2.81–9.96)***
Q2	1.41 (0.87–2.28)	1.60 (0.88–2.90)
Q3	1.62 (1.00–2.62)	1.82 (1.01–3.29)*
Q4	Reference group	Reference group
Weight (m)	0.99 (0.97–1.00)	0.95 (0.93–0.96)***
Chronic obstructive pulmonary disease (yes)	2.64 (1.43–4.87)**	9.77 (5.31–17.99)***
Number of chronic diseases		
0	Reference group	Reference group
1	1.24 (0.87–1.76)	1.31 (0.84–2.05)
2 or more	1.72 (1.16–2.55)**	3.00 (1.90–4.73)***
Medication use		
Ever use corticosteroids > 3 months (yes)	2.04 (0.65–6.40)	2.95 (0.91–9.61)
Smoking		
Never or > 15 years ago	Reference group	Reference group
Former	1.90 (1.19–3.04)**	3.16 (1.86–5.36)***
Current	3.91 (2.31–6.62)***	7.59 (4.29–13.43)***
Physical activity (min/day)		
Q1	1.35 (0.93–1.97)	2.15 (1.40–3.32)***
Q2	1.03 (0.73–1.47)	0.92 (0.59–1.43)
Q3	Reference group	Reference group
Serum 25(OH)D (nmol/L)		
< 25 nmol/L	3.91 (1.38–11.05)*	7.04 (2.30–21.52)***
25–50 nmol/L	2.14 (1.14–4.02)*	2.07 (0.97–4.42)
50–75 nmol/L	1.95 (1.08–3.52)*	1.75 (0.86–3.60)
> 75 nmol/L	Reference group	Reference group
CRP (µg/ml)		
Q1	Reference group	Reference group
Q2	0.95 (0.61–1.48)	1.81 (1.05–3.13)*
Q3	2.12 (1.29–3.47)**	4.83 (2.71–8.63)***
Physical performance (range 0–12)	0.86 (0.81–0.93)***	0.79 (0.73–0.85)***
Grip strength (kgf)		
Q1	3.01 (1.58–5.73)***	6.41 (3.18–12.94)***
Q2	1.78 (1.05–3.01)*	1.87 (1.01–3.47)*
Q3	1.54 (0.96–2.48)	1.03 (0.56–1.87)
Q4	Reference group	Reference group
Perceived self-efficacy (range 12–60)	0.94 (0.91–0.97)***	0.92 (0.88–0.95)***
Pets in the household (yes)^c^	1.20 (0.86–1.68)	1.11 (0.75–1.66)
Dogs in the household (yes)^d^	1.45 (0.83–2.55)	0.74 (0.37–1.51)

*OR* odds ratio, *CI* confidence interval, *PEFR* peak expiratory flow rate, *Q* quartile, *BMI* body mass index, *serum 25(OH)D* serum 25-hydroxyvitamin D, *CRP* C-reactive protein (CRP)

^a^Adjusted for educational level

^b^Adjusted for age

^c^Pet(s) in the household including dogs

^d^Question only asked in persons having a pet

*p < 0.05

**p < 0.01

***p < 0.001

Men who were older (after adjustment for educational level), who had a lower educational level (after adjustment for age), who were in the lowest quartile of height, who had COPD, who had two or more chronic diseases, who were a former or current smoker, who had serum 25(OH)D levels up to 75 nmol/L, who were in the highest tertile of CRP, or who were in the first or second quartile of grip strength, had a higher odds for being in the intermediate as compared with the high PEFR trajectory. They also had a higher odds for being in the low as compared with the high PEFR trajectory, except for men with serum 25(OH)D levels between 25 and 75 nmol/L. In addition, men with a medium educational level (after adjustment for age), who were in the third quartile of height, who were in the lowest tertile of physical activity, or who were in the second tertile of CRP had a higher odds for being in the low as compared with the high PEFR trajectory.

In Table 4, differences in baseline characteristics between trajectories in women are presented after adjustment for age and educational level. Women who were older (after adjustment for educational level), who were in the lowest quartile of height, who had COPD, who had two or more chronic diseases, who reported ever using corticosteroids for longer than three months, or who were a current smoker had a higher odds for being in the low as compared to the high PEFR trajectory. Women who were in the third quartile of weight, who had a higher physical performance score, or a higher grip strength had a lower odds for being in the low versus the high PEFR trajectory.

**Table 4:** Characteristics of low PEFR in women: logistic regression analyses adjusted for age and educational level

Characteristics	OR (95% CI) on low versus high PEFR trajectory
Age (years)^a^	1.04 (1.03–1.06)***
Partner (yes)	0.92 (0.70–1.21)
Educational level^b^	
Lower	1.30 (0.82–2.06)
Medium	0.69 (0.43–1.11)
Higher	Reference group
Height (cm)	
Q1	2.25 (1.44–3.54)***
Q2	1.28 (0.82–1.99)
Q3	1.52 (0.99–2.35)
Q4	Reference group
Weight (m)	
Q1	Reference group
Q2	0.74 (0.48–1.12)
Q3	0.59 (0.38–0.89)*
Q4	0.87 (0.57–1.32)
Chronic obstructive pulmonary disease (yes)	3.58 (2.36–5.42)***
Number of chronic diseases	
0	Reference group
1	0.88 (0.64–1.21)
2 or more	1.54 (1.11–2.15)*
Medication use	
Ever use corticosteroids > 3 months (yes)	2.28 (1.16–4.45)*
Smoking	
Never or > 15 years ago	Reference group
Former	1.36 (0.80–2.31)
Current	2.14 (1.37–3.33)***
Physical activity (min/day)	
Q1	1.06 (0.77–1.47)
Q2	0.92 (0.68–1.26)
Q3	Reference group
Serum 25(OH)D (nmol/L)	
< 25 nmol/L	1.31 (0.57–3.00)
25–50 nmol/L	0.79 (0.39–1.61)
50–75 nmol/L	0.69 (0.34–1.43)
> 75 nmol/L	Reference group
CRP (µg/ml)	
Q1	Reference group
Q2	1.03 (0.69–1.54)
Q3	1.42 (0.96–2.11)
Physical performance (range 0–12)	0.90 (0.86–0.94)***
Grip strength (kgf)	0.89 (0.86–0.92)***
Perceived self-efficacy (range 12–60)	
Q1	1.33 (0.91–1.96)
Q2	1.10 (0.76–1.60)
Q3	1.21 (0.84–1.75)
Q4	Reference group
Pets in the household (yes)	1.08 (0.80–1.46)
Dogs in the household (yes)	0.97 (0.56–1.68)

*OR* odds ratio, *CI* confidence interval, *PEFR* peak expiratory flow rate, *Q* quartile, *BMI* body mass index, *serum 25(OH)D* serum 25-hydroxyvitamin D, *CRP* C-reactive protein (CRP)

^a^Adjusted for educational level

^b^Adjusted for age

^c^Pet(s) in the household including dogs

^d^Question only asked in persons having a pet

*p < 0.05

**p < 0.01

***p < 0.001

## Discussion

This study examined trajectories of PEFR during ageing in older men and women using a mixture model. In men, three declining PEFR trajectories were identified during 13 years of follow-up with prevalence rates of 30%, 46% and 24% for the high, intermediate and low PEFR trajectory, respectively. In women, two declining trajectories were identified with prevalence rates of 62% and 38% for the high and low PEFR trajectory, respectively. All trajectories showed a similar rate of linear decline during ageing and differed mostly with regard to their intercept. In both men and women, age (adjusted for educational level only), height, weight, COPD, number of chronic diseases, smoking, physical performance, and grip strength were significantly associated with the trajectories after adjustment for age and educational level. Partner status, educational level (adjusted for age only), physical activity, serum 25(OH)D, CRP, and perceived self-efficacy were only significantly associated with the trajectories in men, whereas ever use of corticosteroids was only significantly associated in women.

We did not identify other studies examining long-term trajectories of PEFR in older adults using a data-driven method such as LCGA. In a recent systematic review, nine studies were identified examining trajectories of lung function using a data-driven method in the general population [25]. However, all of these studies used spirometry as the primary lung function measurement. In addition, most of these studies were performed in children or young adults. Therefore, we cannot compare these results to our study.

The trajectories observed in the current study mainly differed with respect to their baseline PEFR level, suggesting that differences in PEFR arise earlier in life. In a personal view published in the Lancet Respiratory Medicine, it was described that three phases with regard to lung function can be distinguished: a growth phase with a peak at age 20–25 years, a plateau phase that lasts for a few years, followed by a decline phase due to physiological lung ageing [26]. Depending on numerous genetic and environmental factors, different trajectories may be followed during ageing [26, 27]. The differences in the height of the peak and in the degree of decline are clearly visible in the Tasmanian Longitudinal Health study [28], which is the only study that followed lung function, i.e. FEV_1_, of participants during all three phases [25]. Baseline differences between the trajectories were already visible before the age of 7 [28]. It is likely that similar phases can be identified with regard to PEFR trajectories. This is supported by part of the characteristics identified in our study, such as such as lower educational level, COPD and associated medication use, and smoking, which may also arise in child- or early adulthood. PEFR trajectories during all three phases, should be examined in future studies.

The significant characteristics observed in our study were largely in line with other studies examining predictors or risk factors for impaired lung function [6, 16]. In addition to these well-known characteristics, we also examined partner status, perceived self-efficacy and pets in the household. Having a partner may influence ones behavior and health [18]. In our study, more men and women with a partner were in the high trajectory. However, in women this association was no longer significant after adjustment for age and socioeconomic status. Perceived self-efficacy was lower in men and women in the low PEFR trajectory as compared to the high PEFR trajectories. It may be that perceived self-efficacy is associated with a higher motivation during the test. In women the relation between perceived self-efficacy and the trajectories was explained by age and educational level. We did not find a significant difference for pets in the household. This may possibly be due to opposite effects on PEFR: on the one hand, the hairs of dogs and cats may have a negative effect; on the other hand, having a dog may have a positive effect because of higher activity levels. However, analyzing dogs separately did not change the results. In men, no significant difference was observed with regard to “ever use of corticosteroids longer than 3 months”. This is surprising as we did find a clear difference in prevalence of COPD. However, the prevalence rates of use of corticosteroids was 2.0%, 4.0% and 5.6% in the high, intermediate and low PEFR trajectory, respectively, which is in the expected direction. Possibly, the lack of significance can be explained by a power issue or by an underestimation of the prevalence rates as it may be that recall bias occurred due to self-report of this variable.

It may be considered surprising that older men and women in the worst trajectory showed similar linear decline as in the higher trajectories. One might expect that all persons with COPD and all heavy smokers, for example, are grouped in the worst trajectory. However, considering the prevalence of COPD, we found prevalence rates of 34.6% in the low, 12.1% in the intermediate and 4.8% in the high PEFR trajectory in men, and 19.5% in the low and 5.8% in the high PEFR trajectory in women. This means that there was substantial variation in PEFR trajectories among persons with COPD. It also means that there were many people in the worst trajectory for other reasons than COPD. In a posthoc analysis comparing PEFR decline specifically between persons with and without COPD, we found that the shape and speed of PEFR decline, as well as the variation in trajectories was similar between persons with and without COPD. Yet the starting level of the COPD group was—on average-much lower (data not shown). Therefore, we tentatively attribute this result to already existing differences in PEFR at younger ages, i.e., below the age of 61.

Respiratory function was suggested as one of the measures for assessing vitality [12], one of the domains of intrinsic capacity as proposed by the WHO in 2015. An advantage of PEFR for assessing respiratory function is that it is widely available, cheap, easy to use, and not much of a burden to the patient. The WHO proposes to monitor intrinsic capacity during ageing. In the current study we have shown what trajectories of PEFR are observed in older men and women during ageing, which is important input for developing monitoring strategies. Trajectories in both men and women differed mainly in baseline level of PEFR and not in rate of decline over time, which might indicate that a baseline measurement of PEFR is sufficient to predict someone’s health status in the long-term. However, for monitoring purposes, longitudinal measurements might be informative as a sudden decline in PEFR might indicate a deterioration of someone’s health status. This should be examined further in future studies.

Strengths of the current study are that it was performed in a representative sample of the Dutch older population with thirteen years of follow-up. A limitation is that it is not clear whether our results are generalizable to other countries. Future studies should replicate our findings to study whether trajectories are comparable across countries and ethnicities. Another limitation is that there were relatively few participants in the oldest age categories. As a consequence, a small deviation between observed and estimated average values was observed around the age of 90 years and over, indicating that the fit was less good above the age of 90 years and that the estimates are more uncertain above this age. Finally, a limitation might be that we did not adjust for class uncertainty, which is not possible in SPSS. However, we do not expect a major impact of this as the trajectories observed were clearly distinct from each other, i.e. parallel and not crossing, which was also supported by the highly significant differences in the characteristics.

In conclusion, three long-term trajectories of PEFR were observed in men, while two trajectories of PEFR were observed in women, all showing a similar linear decline during ageing. Men and women in the lower trajectories were older and had a worse health status and lifestyle. Trajectories in both men and women differed mainly in baseline level of PEFR and not in rate of decline over time, which might indicate that one baseline measurement of PEFR is sufficient to give an indication of the trajectory that an older adult is likely to follow. Future studies should examine whether a sudden deviation from this trajectory affects a person’s health status and mortality risk.

## Figures and Tables

**Fig. 2:**
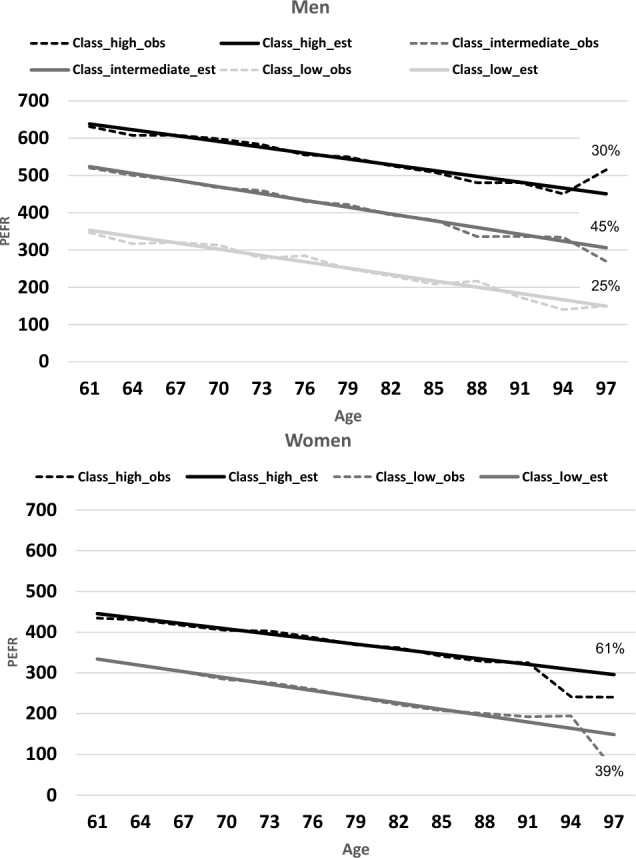
Trajectories of PEFR in men and women taking into account height: sensitivity analysis. *PEFR* Peak expiratory flow rate, *obs* observed values, *est* estimated values. Three classes were identified in men (high, intermediate, low) and two classes were identified in women (high, low). The prevalence of each class was presented as a percentage

## Data Availability

The dataset generated during the current study are not publicly available due to confidentiality, but the data underlying the results presented in this study are available from the Longitudinal Aging Study Amsterdam (LASA). Data of LASA may be requested for research purposes. More information on data requests can be found on the LASA website: www.lasa-vu.nl.

## Appendix

See Fig. 2; Tables 5, 6

**Table 5:** Characteristics of PEFR trajectories in men: univariable analysis

Characteristics	High	Intermediate	Low	p-value
	PEFR trajectory^a^	PEFR trajectory^a^	PEFR trajectory^a^	
Age (years)	70.9 ± 8.3	72.5 ± 8.5	74.2 ± 8.1	< 0.001
Partner (% yes)	90.4	80.0	70.7	< 0.001
Educational level (%)				
Lower	18.1	30.3	36.5	< 0.001
Medium	57.7	52.9	51.5	
Higher	24.2	16.9	12.0	
Height (cm)	175.3 ± 6.4	172.8 ± 6.7	170.9 ± 6.6	< 0.001
Weight (kg)	80.7 ± 10.9	79.1 ± 11.6	73.4 ± 12.0	< 0.001
Chronic obstructive pulmonary disease (% yes)	4.8	12.1	34.6	< 0.001
Number of chronic diseases (%)				
0	43.6	33.0	24.5	< 0.001
1	34.9	35.9	30.8	
2 or more	21.5	31.2	44.7	
Medication use (%)				
Ever use corticosteroids > 3 months (%)	2.0	4.0	5.6	0.18
Smoking (%)				
Never or > 15 years ago	71.6	49.5	36.0	< 0.001
Former	17.4	22.8	26.4	
Current	10.9	27.7	37.6	
Physical activity (min/day)	113.6 [65.7–172.9]	103.9 [49.5–160.5]	76.4 [22.0–147.3]	< 0.001
Serum 25(OH)D (nmol/L)	56.9 ± 18.9	52.4 ± 18.1	48.8 ± 19.0	< 0.001
CRP (µg/ml)	2.3 [1.1–4.8]	3.3 [1.5–7.1]	4.9 [2.5–9.7]	< 0.001
Physical performance (range 0–12)	9.3 ± 2.4	8.2 ± 2.9	7.1 ± 3.2	< 0.001
Grip strength (kgf)	38.0 ± 7.5	34.8 ± 8.0	32.3 ± 8.8	< 0.001
Perceived self-efficacy (range 12–60)	44.2 ± 4.9	42.4 ± 5.2	41.5 ± 5.2	< 0.001
Pet(s) in the household (% yes) ^b^	28.7	31.4	28.3	0.62
Dog(s) in the household (% yes) ^c^	36.1	45.0	28.4	0.06

*PEFR* peak expiratory flow rate, *BMI* body mass index, *serum 25(OH)D* serum 25-hydroxyvitamin D, *CRP* C-reactive protein (CRP).

^a^Presented are the mean ± standard deviation, median [interquartile range] or percentage.

^b^Pet(s) in the household including dogs

^c^Question only asked in persons having a pet

**Table 6:** Characteristics of PEFR trajectories in women: univariable analysis

Characteristics	High PEFR trajectory^a^	Low PEFR trajectory^a^	p-value
Age (years)	71.1 ± 8.3	74.5 ± 8.1	< 0.001
Partner (% yes)	52.6	41.5	< 0.001
Educational level (%)			
Lower	44.3	60.7	< 0.001
Medium	47.5	31.0	
Higher	8.2	8.3	
Height (cm)	161.1 ± 6.2	158.6 ± 6.3	< 0.001
Weight (m)	71.4 ± 12.3	70.4 ± 13.8	0.275
Chronic obstructive pulmonary disease (% yes)	5.8	19.5	< 0.001
Number of chronic diseases (%)			
0	30.8	24.9	< 0.001
1	42.2	33.2	
2 or more	27.1	42.0	
Medication use (%)			
Ever use corticosteroids > 3 months (%)	3.4	7.5	0.011
Smoking (%)			
Never or > 15 years ago	81.7	73.8	0.017
Former	7.9	9.0	
Current	10.4	17.1	
Physical activity (min/day)	169.3 [110.0–238.9]	151.6 [74.5–222.9]	0.002
Serum 25(OH)D (nmol/L)	47.3 ± 17.3	43.2 ± 18.5	0.004
CRP (µg/ml)	2.7 [1.3–5.9]	3.6 [1.6–7.1]	0.016
Physical performance (range 0–12)	7.8 ± 3.1	6.2 ± 3.6	< 0.001
Grip strength (kgf)	21.8 ± 5.0	19.1 ± 4.7	< 0.001
Perceived self-efficacy (range 12–60)	41.6 ± 5.2	40.6 ± 5.2	0.004
Pets in the household (% yes) ^b^	24.8	24.1	0.798
Dog(s) in the household (% yes) ^c^	40.4	34.3	0.329

*PEFR* peak expiratory flow rate, *BMI* body mass index, *serum 25(OH)D* serum 25-hydroxyvitamin D, *CRP* C-reactive protein (CRP).

^a^Presented are the mean ± standard deviation, median [interquartile range] or valid percentage.

^b^Pet(s) in the household including dogs.

^c^Question only asked in persons having a pet
